# The Use of Generic Medications for Glaucoma

**DOI:** 10.1155/2020/1651265

**Published:** 2020-04-07

**Authors:** Andrew J. Tatham

**Affiliations:** Princess Alexandra Eye Pavilion, University of Edinburgh, Chalmers Street, Edinburgh EH3 9HA, UK

## Abstract

The use of generic medicines has grown considerably in recent years providing considerable cost savings. In England, generic items represented 11.7% of prescriptions for glaucoma and ocular hypertension in 2009, increasing to 55.2% of prescriptions in 2018. Generics can be brought to the market quickly and at low cost as manufacturers are not required to repeat animal or clinical research on active ingredients already approved for safety and efficacy. Although there is no regulatory requirement for studies comparing branded and generic eye drops, several randomised crossover studies have been performed comparing branded and generic prostaglandin analogues. While most have shown similar intraocular pressure lowering, studies are of short duration and have not evaluated visual field endpoints. Furthermore, differences in inactive ingredients, pH, viscosity, levels of particulate matter, and degradation over time have been reported. Other potential problems with generic eye drops include differences in bottle design affecting adherence, problems with supply, and the possibility that reduced revenue for innovator companies will lead to reduced investment in new drug development. This article reviews the potential advantages and disadvantages of generic antiglaucoma medications.

## 1. Introduction

Generic medications are defined by the World Health Organization (WHO) as pharmaceutical products intended to be interchangeable with an innovator product, manufactured without a licence from the innovator company and marketed after the expiry date of the patent or other exclusive rights [1]. They are required to have the same active ingredient, route of administration, dosing, and be manufactured to the same quality standards as the reference medication but may have different inactive ingredients and packaging [1, 2]. Generics can only be marketed once the period of exclusivity of innovator product has expired, which is typically 10 years from the date of first authorisation; before a generic medication can be marketed, it must be approved by the appropriate regulatory authority, for example, the European Medicines Agency (EMA) or United States (US) Food and Drugs Administration (FDA) [3]. Regulatory authorities conduct rigorous reviews to ensure generic drugs are of a high standard, conduct inspections of manufacturing facilities, and monitor drug safety after products are brought to market [4].

In recent years, the use of generic medicines has grown considerably, driven by their often substantially lower price compared to branded products. Generic items accounted for only 19% of prescription drugs sold in the US in 1984, increasing to 43% in 1996, and 89% in 2017 [5]. This has resulted in significant savings for healthcare systems, with an estimated saving in the US of $1.67 trillion between 2007 and 2016 [6]. The use of generics has also increased in ophthalmology, especially in the medical treatment of glaucoma. In England, generic items represented 11.7% of prescriptions for glaucoma and ocular hypertension in 2009, increasing to 55.2% of prescriptions in 2018 [7]. The first generic prostaglandin analogue (PGA), latanoprost, became available in early 2011.

There are however potential downsides to the use of generic medicines, including inconsistences in packaging and bottle design between products, differences in inactive ingredients which may cause unexpected issues with tolerability, and the likelihood that reduced revenue for innovator companies will lead to reduced investment in new drug development. In addition, some professionals and members of the public hold the view that generics are less effective and of poorer quality than branded alternatives, which may reduce acceptance of generic drugs. As much debate surrounds the use of generic medicines, the aim of this article is to review of the use of generics for glaucoma and provide a balanced appraisal of potential advantages and disadvantages.

## 2. Methods

**Figure 1:**
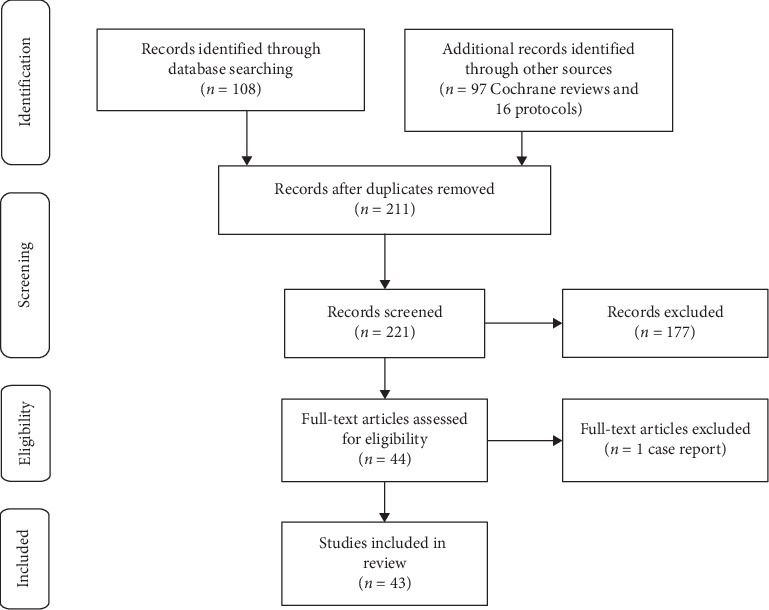
Preferred reporting items for systematic reviews and meta-analyses (PRISMA) diagram.

A PubMed database search was performed on 16th December 2019 using the following search terms: ((glaucoma[Title/Abstract]) AND generic[Title/Abstract]) AND (“1900/01/01”[Date - Publication]: “2019/12/16” (Date—Publication)). The Cochrane Library was also searched for meta-analyses or systemic reviews containing the keyword “generic” in their title or abstract. The PubMed search yielded 108 items, while 97 Cochrane reviews and 16 Cochrane protocols were identified (Figure 1). Article titles and abstracts were manually reviewed and of the 221 records screened, 178 were excluded as they were not deemed to relate to the use of generic medications in glaucoma. Though Cochrane protocols were identified proposing reviews of generic versus branded antiepileptic drug monotherapy in epilepsy (18th Sep 2015) and clozapine (generic versus branded) for schizophrenia (24th Jan 2019), no completed reviews were found and no Cochrane reviews or protocols were identified relating to generic ophthalmic medications. The full texts of the remaining 44 records were examined and one was excluded as it related to a single case report. Of the remaining articles, 15 primarily focused on cost analysis of glaucoma medications, 11 were experimental laboratory studies, for example, examining drug composition or bottle designs, 6 were randomised trials or switch studies, there were 4 reviews, and 7 studies of other design, for example, examining trends in prescribing patterns or comparing adherence between generic and branded medications.

### 2.1. Efficacy

Although there have been several meta-analyses conducted in areas outside ophthalmology showing no difference in outcome between branded and generic medications, including for cardiovascular drugs, antiepilepsy medications, and some antibiotics [8–10], our search found no meta-analyses or systematic reviews examining the efficacy of generic compared to branded antiglaucoma medications. The lack of studies is likely due to the fact that they are not required by regulatory authorities for market authorisation.

The World Health Organization (WHO) has developed global standards and requirements for the regulatory assessment, marketing authorisation, and quality control of generic medications [11]. These standards specify that generic products should fulfil three sets of criteria relating to (1) manufacture and quality control; (2) product characteristics and labelling; and (3) therapeutic equivalence, with assessment of equivalence normally requiring in vivo studies. Generic medications can be brought to market quickly as manufacturers are not required to repeat animal or clinical research on active ingredients already approved for safety and efficacy. However, generic products must have pharmaceutical equivalence to the innovator product, meaning they contain the same amount of the same active substance(s) in the same dosage form, as well as bioequivalence, meaning bioavailability is within an acceptable limit. It is important to emphasise that pharmaceutical equivalence does not necessarily equate to bioequivalence as differences in excipients and/or the manufacturing process can lead to differences in absorption. Bioequivalence studies usually involve an assessment of rate and extent of absorption using the plasma concentration time curve. For example, the European Medicines Agency requires characteristics such as maximum serum concentration and AUC0-t (area under the plot of drug concentration over time curve from drug administration) to be within 80 to 125% of the reference product [12]. Classic bioequivalence studies cannot be performed on locally acting drugs such as eye drops as their active ingredients are not found in measurable quantities in the bloodstream and bloodstream levels are not related to efficacy [13]. Therefore, some products may be considered bioequivalent without the need for bioequivalence studies. The WHO list several examples of potentially exempt products including parenterally administered medications containing the same concentration of active substance with the same excipients; orally administered medications containing the same concentration of active substance, that do not contain an excipient known or suspected to affect absorption of the active ingredient; and, most relevant to this review, ophthalmic products prepared as aqueous solutions containing the same active substance in the same concentration and essentially the same excipients in comparable concentrations [11].

While there is no requirement for manufacturers to conduct head to head clinical studies of generic and branded topical ophthalmic products, several comparison studies have been published. The first study comparing the efficacy of branded Xalatan and generic latanoprost was published in 2007 [14]. Narayanaswamy and colleagues reported the results of an open-label randomised crossover study in which 30 participants received each treatment for a 12-week period. A larger percentage reduction in intraocular pressure (IOP) was observed when using Xalatan compared to the generic (38.66 ± 10.29% versus 25.42 ± 5.98%). In patients switching from generic latanoprost to Xalatan, there was an additional 4.3 ± 8.76% reduction in IOP, compared to an average 8.86±17.76% increase in IOP when switched from Xalatan to generic latanoprost. There was also a lower incidence of conjunctival hyperaemia and ocular irritation assumed to be due to higher amounts of particulate matter observed in the generic.

In contrast, subsequent studies have largely found similar efficacy between branded and nonbranded prostaglandin analogues (PGAs) [13–15]. A large double-masked study of 184 patients in Italy found noninferiority of generic latanoprost to Xalatan, with no difference in adverse events [15]. Similarly, Golan and colleagues conducted a randomised crossover study comparing Xalatan and a generic latanoprost, with patients masked to the medication they were receiving [16]. There were no significant differences in IOP lowering between groups but more ocular surface disease-type side effects were reported when using the generic. The differences in study results may reflect the large number of different generics available. The FDA hosts an “Orange Book” of approved drug products with therapeutic equivalence evaluations that can be used to verify whether particular generics have been approved for use in the US [17]. A search in December 2019 revealed 9 generic versions of latanoprost 0.005%.

Diagourtas and colleagues recently compared two generic PGAs available in Greece to branded latanoprost in 60 patients who had never received antiglaucoma treatment [13]. Although the study lasted only 16 weeks and did not include a crossover phase, patients were masked to the medication they were receiving. The generic drops produced similar IOP lowering compared to Xalatan, with percentage IOP reductions from 30.34 to 32.06%. The first study comparing the effectiveness of branded and generic travoprost was published in 2019 [18]. This prospective study of 70 patients, randomised patients to either branded Travatan *Z* (Alcon) or a generic travoprost (Sandoz Inc). Intraocular pressure was measured at baseline and after 3 weeks of treatment, after which patients switched medication, with a further IOP assessment at week 6. The IOP lowering effect of generic travoprost was found to be equivalent to Travatan. A questionnaire was used to assess tolerability, and this was found to be similar between formulations; however, this study had the disadvantage of patients not being masked to the study medications.

Although previous studies have compared the effect of branded and generic medications on ocular surface disease symptoms, their primary endpoint has been IOP. It is desirable to obtain data regarding more clinically relevant endpoints such as visual function and other patient reported outcomes. The only study identified in our literature search that examined a non-IOP endpoint was that of Kim and colleagues who used US commercial medical claims data to compare the hazard of needing a second glaucoma medication or surgical intervention for glaucoma in patients using generic latanoprost or a branded PGA [18]. The study identified 6,317 patients with primary open angle glaucoma using generic latanoprost and 3,703 using branded PGAs. Use of generic latanoprost was associated with a reduced hazard of undergoing a glaucoma procedure (HR = 0.72, 95% CI 0.62–0.84) but not with needing a second glaucoma medication (HR = 0.95, 95% CI 0.87–1.03), likely due to the reduced cost of generic medications leading to improved adherence.

### 2.2. Costs

The major advantage of generic over branded medications is lower cost. Over the last 20 years, there has been an increase in the number of antiglaucoma medications prescribed per head of population, with an associated increase in costs; however, an increase in the use of generic medications has slowed growth in expenditure. In England, between 2000 and 2012 there was a 67% increase in prescriptions for glaucoma issued in primary care, likely driven by improved case finding and an ageing population [19]. This was associated with an 88% increase in medication costs, from £55.2 million annually in 2000 to £103.7 million annually in 2012 [19]. Between 2009 and 2018, the number of items prescribed grew from 1,382 per 10,000 people to 1,668 per 10,000 people [7]; however, prescribing costs remained relatively stable, largely due to increased use of generics. The proportion of generic medications prescribed during this time increased from 11.7% in 2009 to 55.2% in 2018, with the contribution of generic medications towards the total cost of glaucoma prescribing increasing from 4.4% to 37% [7].

There is though large variation in generic penetration between countries, likely in large part due to differences in price regulation and payment systems. European generic medicine pricing tends to follow either a free market approach, where manufacturers are relatively free to set prices, or a price-regulated system, where prices are set by law [20]. Penetration of generic medications is more successful in countries that permit free pricing of medicine as manufacturers of originator medicines tend to charge premium prices, attracting entry of generic products, whose manufacturers have room to profit while still undercutting the cost of the branded product. In countries with price regulation, the price of the originator medicine is driven down discouraging entry of generics and restricting price competition after patent expiry. In addition, countries with price-regulated systems often link the price of generic products to a reference price related to the branded equivalent, enabling manufacturers of originator medicines to lower prices to drive generic medicines out of the market [20].

Despite lower cost being a major advantage of generic medications, the medication prices are subject to fluctuation. For example, in 2018 there was a temporary 8-fold increase in the price of generic latanoprost in the UK due to a shortage [7]. The reasons for medication shortages are complex, but the FDA recently convened a drug shortages task force to examine the problem [21]. One hundred and sixty-three drugs were identified that went into shortage between 2013 and 2017, and these were compared to similar medicines not in short supply. Shortage drugs were more likely to be low priced and financially unattractive for manufacturers, with shortages often due to disruption in the supply chain. In many cases, manufacturers had discontinued the production of medications due to loss of profitability. The task force highlighted that driving down cost to the lowest possible price disincentivises investment by manufacturers which may increase the risk of manufacturing problems or prompt them to leave the market, and shortages were compounded by logistical and regulatory hurdles being too great for other companies to increase production during a shortage [21]. Drug shortages can have severe consequences; for example, a 2011 shortage of norepinephrine in the US was significantly associated with an increase in mortality in patients with septic shock [22]. The European Medicines Agency publishes information on specific medicine shortages affecting one or more European Union member states and includes links to national shortage registers [23].

It is also important to consider the possibility that cheaper medication costs may not automatically translate to cheaper overall costs. Dubois summarised several scenarios where use of generic eye drops may not automatically lead to a cost saving [24]. For example, if the switch to a generic leads to reduced adherence due to difficulty using a new bottle design, if the patient beings running out of medication early due to the generic bottle dispensing too large volume of medication, or if a difference in inactive ingredients leads to a higher rate of ocular surface disease. The use of generic medications may also increase the risk of dispensing errors, particularly with fixed dose combination medications, where one medication could be omitted from the repeat prescription or missed due to a dispensing error.

### 2.3. Tolerability and Differences in Formulation

The presumption that generics are equal to branded medications because the active ingredients are the same is also not necessarily true. Regulatory authorities require generic and branded medications to contain the same active ingredient but excipients may vary. Excipients are heterogenous inert pharmaceutical ingredients used in product formulations, for example, thickening agents and buffers. In most cases, they have limited or no pharmacological activity, but they can influence drug stability and bioavailability and these differences have the potential to affect efficacy [25, 26]. Kolko and colleagues examined the physical properties of 5 generic latanoprost solutions and found substantial differences to the branded version [26]. The pH of branded latanoprost was markedly lower than the generic products, and there was significant variation in viscosity. The difference in observed pH was unexpected as the advertised label pH of generic latanoprost is typically similar to Xalatan [27].

Kahook and colleagues also examined the composition of generic and branded PGAs and using mass spectroscopy found differences in the quantity of active ingredients and excipients [28]. Although the FDA requires concentrations of active ingredients to be within 10% of the labelled value, some generic medications had concentrations exceeding this [28]. Generic medications also had higher levels of particulate matter, the origin of which was presumed to be either contaminants, precipitates of active ingredients, or material from the eye drop container. Latanoprost can degrade at high room temperature, and there may be differences in degradation between formulations. Kahook found some bottles of generic latanoprost had a significant decrease in latanoprost over time, with loss of more than 10% of active ingredient after exposure to temperature levels at the higher end of their labelled indication (25°C for 30 days), raising questions about the stability of generic formulations. Degradation of benzalkonium chloride (BAK) was also observed. As the reduction in IOP with latanoprost is dose dependent, with an optimal concentration of 0.005% or 0.006%, changes in concentration due to instability may affect efficacy and may require higher concentrations of active ingredients at baseline to counter degradation.

Narayanaswamy also found generic latanoprost to have a higher pH and increased particulate matter compared to branded Xalatan, which was proposed to be a reason for lower therapeutic efficacy observed in their study [14]. The authors concluded that caution should be exercised when switching from branded Xalatan to a generic due to potential changes in efficacy. Velpandian and colleagues examined the concentration of latanoprost in generic medications available in India [29]. The latanoprost content varied from 90 to 330% of the labelled claim, compared to 97% for branded latanoprost, a much greater degree of variability than in the US generics studied by Kahook [28]. There were also differences in degradation of latanoprost due to UV light and heat, made to simulate patient usage.

Leitritz and colleagues examined the concentration of latanoprost and BAK in 23 generic latanoprost formulations [30]. Although the pH of the generic drugs was similar to Xalatan (median 6.78, min 6.62, and max 6.81), all products contained less than the supposed 50 ug/mL of active ingredient. In contrast, most had higher concentrations of BAK than the original drug, with a mean 5.45% greater concentration (range -2.5% to 11.5%). Hallaji et al. also examined the preservative concentration of generic glaucoma eye drops compared to respective brands [31]. Most generics and branded products were found to have the same preservative. High performance liquid chromatography was used to measure the concentration of BAK in branded Xalatan 0.02% and 4 generic versions of latanoprost and found none varied by more than 10% from the concentration found in the branded product. The main exception was a slight difference in the sodium chlorite concentration of the preservative in Alphagan P 0.15% w/v (Allergan) and the generic equivalent [31].

### 2.4. Adherence and Ease of Use

A potential problem of generic eye drops is the difference in bottle design between manufacturers, which may adversely adherence [32]. Many patients with glaucoma struggle to correctly instil their eye drops and this problem likely to be worsened if they receive different bottles over time. Whereas the packaging of innovator products is carefully designed and evaluated in clinical trials, aiming to identify issues with bottle design, the same is not true of generic eye drops. Several studies have shown considerable variation in the force required to squeeze different bottles and successfully release a drop [26, 33, 34]. For example, Kolko and colleagues reported a considerable difference in force was needed to expel drops from a bottle of Xalatan compared to different generic latanoprost bottles, with Xalatan requiring the least pressure [26]. This is likely to be particularly important for patients who struggle to instil drops, such as those with arthritis or reduced strength.

A study examining patient experiences of the transition from Xalatan to generic latanoprost reported patients found drops from Xalatan bottles easier to instil, more comfortable in the eye and easier to open [35]. In 20% of patients, generic bottles failed to last for a full month. This study was though unmasked and may have been influenced by patients' perceptions of a preference towards a branded or familiar product.

Differences in bottle design also contribute to differences in drop size and volume and number of drops per bottle, consequently affecting the quantity of active ingredient delivered to the eye [26, 33, 34, 36]. A study by Mammo and colleagues found differences in the volume of eye drops delivered by a branded topical beta-blocker medication compared to generic versions, while others have reported similar findings with generic and branded PGAs [26, 34].

Differences in bottle design also complicate the use of drop delivery aids. The drop delivery aid for Xalatan (Xal-ease) does not fit generic versions, except the Pfizer generic, and therefore there is a danger that patients switched from branded to generic drops, or those switched between generics, may be unable to use their normal delivery aid [27]. Pharmacists frequently have little control over which generic product they stock or choose the cheapest generic available. Market forces can lead to frequent changes in the generic medications stocked, and patients who have been used to using the same medication for several years may find it confusing when changes are made.

Given the importance of eye drop bottle design to adherence and therefore efficacy, in addition to the effect on stability of the active ingredient, it would seem prudent that stricter regulation of bottle design be considered. For example, if all PGAs were housed in bottles of the same shape and rigidity, it would be easier for patients to use generic products made by different manufacturers, and there would be a greater likelihood of consistent drug delivery. Bottle design is intricately linked to bioavailability and therefore should be considered by the regulatory authorities when evaluating generic medications for market entry. In 2013, the European Medicines Agency released a concept paper on the development of product-specific guidance on demonstration of bioequivalence for generic medicines and subsequently several product-specific guidelines have been produced [37]. Product-specific guidelines for antiglaucoma eye drops may be worthwhile, especially if they stipulated consistency in bottle design.

Though differences in bottle design may be a barrier to adherence, in many healthcare systems cost may be the greater barrier, with the result that switching from branded to cheaper generic medications improves adherence. A previous study in the US reported 41% of patients had difficulty paying for their glaucoma medications [38]. Stein and colleagues examined the impact of the introduction of generic latanoprost on adherence in a large US managed care network [39]. Adherence rates were examined for 18-month periods before and after the introduction of generic latanoprost. When only branded PGAs were available, a subset of patients were noted to have poor adherence, which considerably improved when they were switched to generic latanoprost. This group obtained higher levels of adherence than those who had been maintained on branded PGAs. Although it was not possible to determine the reason for improved adherence with a switch to generics, it was thought to be due to lower costs to patients.

### 2.5. Perceptions of Generic Medications

A number of studies examining attitudes and awareness of generic medications among healthcare professionals and members of the public have shown a lack of confidence in generic prescribing. A review article found 21 publications examining the topic of physician perception of generic medications and 36 reports on patient opinions [40]. The authors concluded that although opinions of generic medicines have improved over the years, some mistrust remains, particularly among patients. Patients tend to prefer branded medications and many do not consider generic medicines equivalent to branded products. There is also a belief that branded products have greater potency and fewer side effects. In several studies, patients seemed to be more accepting of generics for treatment of minor illnesses but preferred branded medicines for more serious problems. Patients also reported that variability in packaging and appearance made it more difficult to keep track of their medications, and some patients were found to be taking two or more equivalent medications due to differences in packaging [41].

It is likely that many of the negative perceptions of generic medications among patients are due to insufficient knowledge and information, and therefore healthcare professionals have an important role to communicate information about the equivalence of generic formulations, which is likely to improve confidence and adherence. A short explanation has been shown to improve the likelihood that a patient will accept a generic [42], and it is the authors view that a switch to a generic should not take place without the patient being informed. Acceptance of generics appears to be higher in patients with higher levels of education, while patients from lower socioeconomic demographics tend to have greater mistrust of generics, although this has not been a universal finding [40].

In the US, ophthalmologists have a higher rate of branded medicine prescribing than any other medical specialty, suggesting greater confidence in branded drugs; however, the use of generic medicines is increasingly rapidly [43]. The large increase in use of generic antiglaucoma medications suggests ophthalmologists now accept the use of generics and have changed their prescribing habits; however, change may also be driven by prescribing or dispensing rules. Changes to treatment guidelines are also likely to have contributed; for example, in the United Kingdom, the National Institute for Health and Care Excellence (NICE) glaucoma guidelines recommend that generic medication be considered first choice treatment [44]. Clinicians undertaking a large scale shift to generic prescribing should however be aware of the potential problems with the use of generic eye drops. Branded and generic medications are not identical. and patients should be informed of potential changes to their medication. It is also important to emphasise to patients that generics are authorised off patent versions of branded medications as not all patients appreciate the difference between generic and counterfeit medicines [45].

## 3. Conclusion

The bioavailability of medicines administered as eye drops is influenced by more than the concentration of the active ingredient, with bottle design one of the most important factors. Stricter regulation of bottle design should be considered to improve consistency of drug delivery, perhaps through product-specific guidance on demonstration of bioequivalence. Although several randomised crossover studies have shown similar efficacy of branded and generic prostaglandin analogues, this finding has not been universal and pharmacological studies have shown differences in the composition and properties of branded and generic antiglaucoma medications.

Generic medicines are here to stay, and unless new classes of superior antiglaucoma medications become available, generics will become increasingly common as older medications come off-patent. The shift to generic prescribing has a great potential for reducing healthcare-related costs; however, it is important that the limitations of generic medications are understood and addressed.
